# Novel 3D-flower shaped KTaO_3_ perovskite for highly efficient photocatalytic and H_2_ generation ability

**DOI:** 10.1038/s41598-022-14590-3

**Published:** 2022-06-24

**Authors:** H. N. Sumedha, M. Shashank, Sergio R. Teixeira, B. M. Praveen, G. Nagaraju

**Affiliations:** 1 Department of Chemistry, Institute of Engineering and Technology, Srinivas University, Mukka, Mangalore, Karnataka 574146 India; 2 Centre for Incubation, Innovation, Research and Consultancy (CIIRC), Jyothy Institute of Technology, Thataguni, Bengaluru, Karnataka 560082 India; 3 Energy Materials Research Laboratory, Siddaganga Institute of Technology (Affiliated to VTU, Belagavi), Tumakuru, Karnataka 572103 India; 4 Department of Studies and Research in Industrial Chemistry, School of Chemical Science, Kuvempu University, Shankaraghatta, Karnataka 577451 India; 5 Laboratory of Thin Films and Nanostructure Fabrication (L3Fnano), Institute of Physics, Universidade Federal Do Rio Grande Do Sul, UFRGS, Porto Alegre, Brazil

**Keywords:** Materials science, Nanoscience and technology

## Abstract

Treatment of industrial wastewater is one of the biggest challenges that mankind is facing today to prevent environmental pollution and its associated adverse effects on human health. Environmentalists across the world have given a clarion call for dye degradation, wastewater treatment and their effective management in our surrounding habitats. Despite significant progress in the development of new water treatment technologies, new materials haven’t matured enough for large scale industrial applications. Hence, the development of new scalable and sustainable multifunctional materials having the potential to treat wastewater and generate energy is the need of the hour. In this direction, novel 3D-flower shaped KTaO_3_ (3D-F-KT) material has been synthesized using areca seed powder as a green fuel. This new material has been successfully applied for the treatment of industrial wastewater contaminated with Rose Bengal. The efficiency of the material was analysed using several parameters like catalytic loading, dye concentration, kinetic and scavenging experiments, photostability, effect of co-existing ions and recyclability. In addition, the material was subjected to optical studies and H_2_ generation, making it a highly versatile multifunctional material, exhibiting a degradation efficiency of 94.12% in a short span of 150 min and a photocatalytic H_2_ generation efficiency of 374 µmol g^−1^ through water splitting. With an immense potential, KTaO_3_ presents itself as a multifunctional catalyst that can be scaled up for a variety of industrial applications ranging from wastewater treatment to energy generation and storage.

## Introduction

One of the major sources of water pollution is the rejected wastes from textile industries. The dissolved dye stuffs in water bodies can cause serious problems to aquatic animals and human life, in addition to various chemical and biological changes that occur during the utilisation of dissolved oxygen^1^. At present, textile wastes are being cleaned using various chemical, biological and physical processes. Conventional techniques like sedimentation, oxidation by chemical methods, filtration and coagulations have many drawbacks due to the formation of various by products, and the associated energy and cost intensive methods^2,3^. Hence, nanoparticles with high photocatalytic properties are being explored as a solution to eliminate harmful pollutants from water bodies. Semiconductor-based metal oxide nanoparticles (MNPs) are capable of removing organic pollutants from wastewater streams and exhibit good catalytic activity^4–6^. The higher catalytic activity is due to the interaction of light rays (photon) and the catalyst that yields CO_2_ and H_2_O^7–9^.

Over the past few years, tantalates have garnered significant research interest because of their potential in a wide range of applications e.g., wastewater treatment, air adsorbents, energy storage, production of hydrogen, etc. Perovskite structures of tanatalates such as NaTaO_3_, KTaO_3_, and LiTaO_3_ have been recently reported to exhibit good photocatalytic property. In particular, potassium tantalate (KTaO_3_) is an outstanding material on account of its excellent dielectric properties and perovskite cubic structure at all temperatures. The conduction band with the Ta (5d) orbital observed at higher negative position is one of the reason for its excellent behaviour during photocatalytic reactions^10,11^. Such photocatalysts also tend to possess abilities to act as electrocatalysts in the generation of hydrogen through the process of water splitting, which is known as green hydrogen. Among the renewable and non-renewable sources of energy, hydrogen production via renewable processes is perceived as one of the cleanest forms of energy, as the non-renewable fuel sources pose significant challenges in terms of sustainability and environmental impact. The surge in the energy requirements across the globe has created a need to find cost-effective alternatives to the currently used expensive catalysts such as platinum (Pt) and iridium (Ir). In this direction, the uses of solar energy to obtain pure hydrogen from water with the help of photoelectrochemical and photocatalytic processes have attracted significant interest^12^. Production of hydrogen by splitting water using sunlight is a promising way to convert solar energy into chemical energy. This type of conversion is more sustainable compared to other processes as it needs only water, catalyst and light^13,14^. Therefore, choosing a convenient photocatalyst that degrades pollutants in water and also exhibits superior water splitting abilities is crucial and highly essential. ^7,15,16^. In addition, due to its chemical stability, photoactivity and nontoxicity, perovskite-structured tantalates have now been effectively used as an essential class of photocatalysts in both the mineralization of organic contaminants and water splitting. Various work has been done so far in order to achieve good photocatalyic activity, Tantalum is used as a doping agent (in different proportions i.e 3, 5, 7 mol% Ta @Cds) in photocatalytic degradation and achieved excellent photocatalytic activity for methylene blue dye^17^. Later Adriana zaleska et al. used KTaO_3_:CdS:MoS_2_ composites to degrade organic pollutants like phenol and toluene. Meanwhile, In-situ growth of Au on KTaO_3_ sub micron cubes were prepared by Jin Wang group to degrade p-nitrophenol pollutant^18^. Changao L et al. synthesised novel BiOl/KTaO_3_ heterostructure for photocataytic degradation of Rhodamine B pollutant by visible light irradiation^19^. The photocatalytic performance of perovskite alkali metal tantalates (NaTaO_3_, KTaO_3_) has been studied in detail and different approaches of their preparation have been examined. These materials are abundant, non-toxic and have a higher negative conduction band edge than the H^+^/H^2^ energy levels^20–22^. Here, we have synthesised KTaO_3_ by combustion method using areca seed as a green fuel, and analyzed the structural and morphological characteristics of the synthesised material using various analytical, spectroscopic and microscopic techniques. In addition, the kinetics, mechanism of photodegradation, and water splitting ability of the material for the generation of hydrogen have been reported in detail, providing new dimensions for the development of such novel materials and extension of their applications beyond wastewater treatment.

## Materials and methods

### Preparation of areca seed powder

Areca seeds were sourced from an agricultural area in Sringeri, Chikmagalur District, Karnataka, India. The plant material was initially washed with distilled water several times and dried in shade for 2–3 days. The obtained dried seeds were powdered into uniform sized particles using an electric mixer (Philips Mixer Grinder, 750 W) and were stored at room temperature until further use.

### Synthesis method

3D flower-like KTaO_3_ (3D-F-KT) nanostructures were prepared by an eco-friendly green combustion process using areca seed powder as the fuel. Tantalum (V) oxide (Ta_2_O_5_) and potassium hydroxide (KOH) were procured from Sigma Aldrich (AR) and used without any further purification. 0.4 g each of Ta_2_O_5_ and areca seed powder were mixed thoroughly and ground in an agate mortar for 15 min. The obtained mixture was added to a crucible containing 1.4 M KOH solution and was stirred for 15 min. The crucible was kept in a pre-heated muffle furnace at 500 ± 10 °C for nearly 10 min and was later calcined at 700 °C for 4 h in static air. A fine milky white powder was finally obtained. The synthesis of 3D-F-KT nanostructures was repeated with different concentrations of areca seed such as 0.2 and 0.8 g respectively. The obtained product was stored in an air tight container for further use.

### Characterization

The particle size and morphological features were studied using scanning electron microscopy (Neo-Scope JCM-6000PLUS system) and transmission electron microscopy (JEOL-JEM 2100). The phase purity and crystal structure were determined by the X-ray diffraction patterns and were acquired using Rigaku Smartlab X-ray diffractometer (Cu-Kα, λ = 1.5406 Å) operating at 40 kV and 30 mA. The infrared spectrum was recorded using a Fourier Transform Infrared Spectrometer (Bruker Alpha P) in the range of 400 to 4000 cm^−1^. The photoluminescence (PL) of the samples were recorded with an Agilent Technology-Cary-60 and their optical properties were analysed using LABINDIA technologies UV 3092 spectrophotometer. The specific surface area and pore size of the catalyst was studied using Quanta Chrome Nova 2200E—BET Surface Area Analyser. The different states and valence of material was analysed by X-ray photoelectron spectroscopy (XPS) using Thermo Fisher ESCALAB Xi^+^ instrument. The radicals formed during degradation process was confirmed and analysed by ESR-JEOL, Japan spectrometer.

## Results and discussions

### Powder X-ray diffraction (P-XRD)

**Figure 1:**
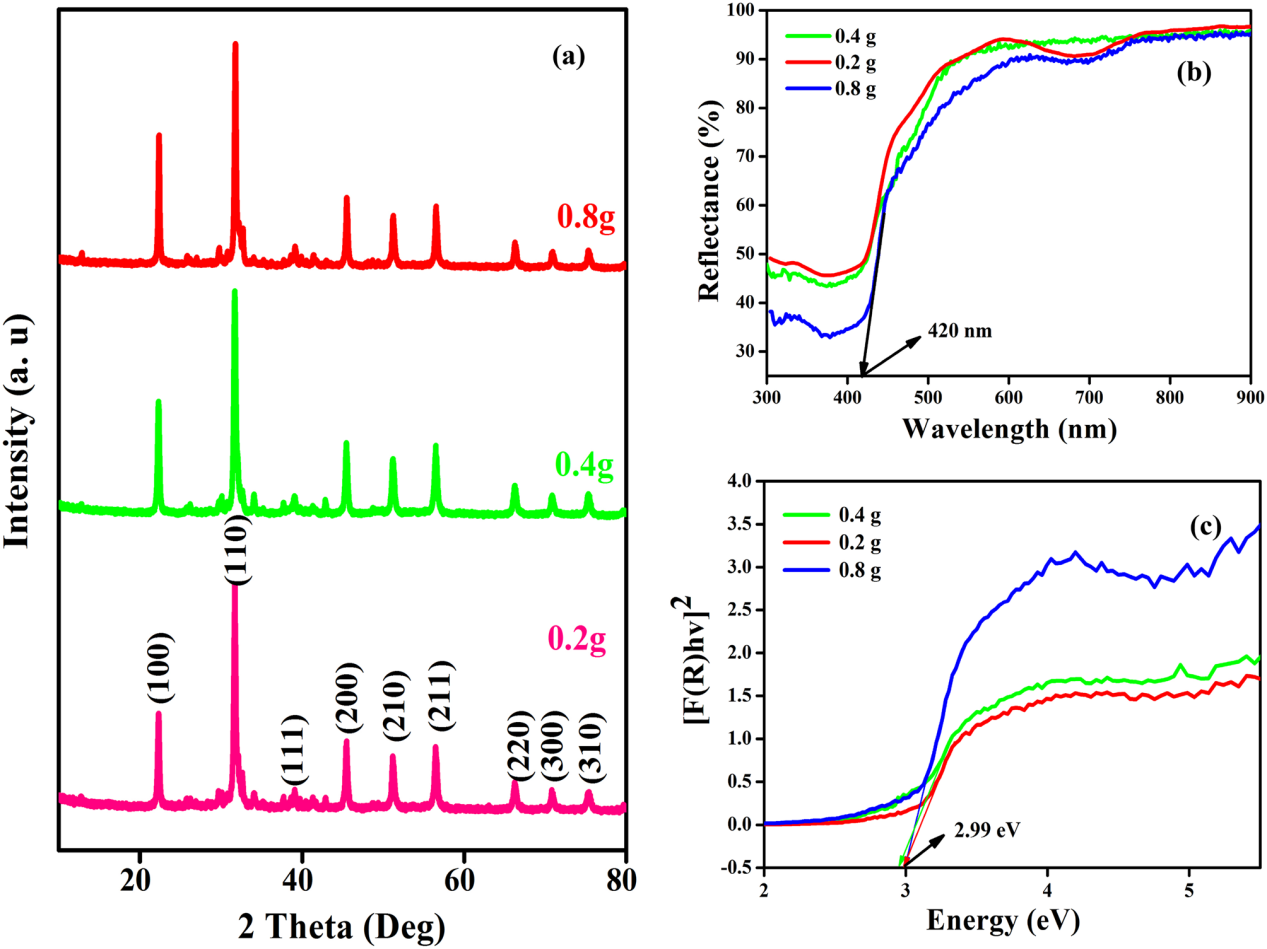
(**a**) Intense XRD peaks of 3D-F-KT NPs prepared by 0.2, 0.4, 0.8 g concentration of areca seed powder. (**b**) Diffuse reflectance spectra showing absorption towards visible region on increasing areca fuel weight of 3D-F-KT. (**c**) Controlled Direct band gap energy (E_g_) of 3D-F-KT NPs achieved by increasing fuel ratio.

Figure 1 shows the XRD patterns of the 3D-F-KT nanostructures synthesized using areca seed powder with different concentrations (0.2, 0.4, 0.8 g). All the peaks (100), (110), (111), (200), (210), (211), (220), (300), (310), (311), (222), (320) match well with the JCPDS Card No.38–1470 having lattice constants of a = b = c = 3.9883 Å, α = β = γ = 90°, space group = Pm-3 m and space group number = 221. The average crystallite size of 3D-F-KT nanostructures (0.2, 0.4, 0.8 g of areca seed powder concentration) was calculated using the following Debye-Scherer’s equation and was found to be 29.07, 26.89, and 33.12 nm respectively.

1 $$D=\frac{K\lambda }{\beta Cos\theta }$$

where K is the crystallite shape constant (0.89), λ is the wavelength of X-ray Cu-Kα radiation (1.5406 Å), β is the Full width at half maximum (FWHM) and θ is the glancing angle. The strong and narrow width of the peaks indicates the high purity and crystallinity of 3D-F-KT nanostructures. Therefore, from the above data it can be inferred that the fuel (areca seed powder) could be responsible for the variation in particle sizes of 3D-F-KT nanostructures^23,24^.

### Diffuse reflectance spectra (UV-DRS)

The diffuse reflectance spectrum was used to observe the band gap of 3D-F-KT nanostructures, which displayed an intense band at 420 nm, indicating the absorption of the host lattice. It can be clearly seen that the addition of more than 50 wt% of the fuel significantly affects the optical absorption properties of 3D-F-KT nanostructures and exhibits an enhanced visible light absorption towards the longer wavelength. Hence, to determine the band gap (E_g_), Kubelka–Munk (K–M) theory was adopted from the diffuse reflectance spectra. The tangent interception of the [F(R)_∞_hν]^1/2^ plots versus photon energy (hν) has been shown in Fig. 1b,c. The photon energy (hν) and Kubelka−Munk function F(R_∞_) were calculated using the following equations.

2 $$F\left({R}_{\infty }\right)=\frac{(1-{R}_{\infty })}{2{R}_{\infty }}$$

3 $$h\nu =\frac{1240}{\uplambda }$$

where R_∞_ is the reflection coefficient and λ is the absorption wavelength. The energy gap was found to be ~ 2.99 eV for the 3D-F-KT material.

### Fourier Transform Infrared Spectra (FTIR)

**Figure 2:**
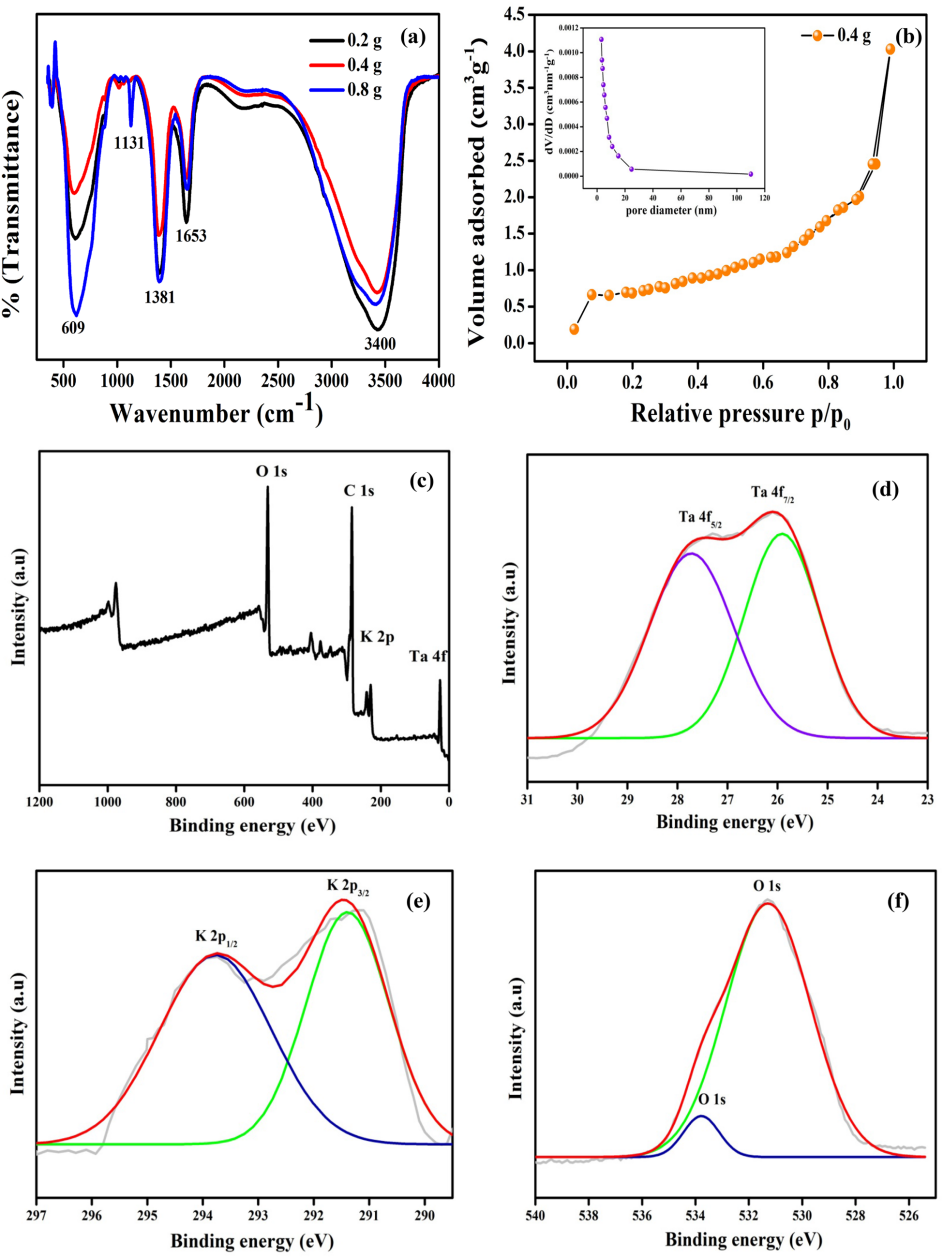
(**a**) FTIR spectra of 3D-F-KT NPs showing intense Ta-O linkage (609 cm^−1^) along with C=C vibration (1653 cm^−1^) and alcoholic C–OH bending (1381 cm^−1^). Another peak at 1131 cm^−1^ is due to C–O stretching, which is caused by adding excess amount of areca seed powder. (**b**) BET specific surface area, pore volume and average pore diameter of 3D-F-KT NPs (Inset: Pore diameter measurements). (**c**) XPS survey of 0.4 g concentration of areca seed powder 3D-F-KT. (**d**) Ta 4f. (**e**) K 2*p* (**f**) O 1*s*.

FTIR spectra of 3D-F-KT nanostructures were recorded from 400 to 4000 cm^−1^ as shown in Fig. 2a. An intense broad peak of Ta-O was observed at 609 cm^−1^ for the three different concentrations (i.e., 0.2, 0.4, 0.8 g) of 3D-F-KT nanostructures synthesized using areca seed powder. It was clearly observed that the carbon content present in the prepared material corresponds to the C=C vibration of the aromatic ring at 1653 cm^−1^ and the hydroxyl C–OH bending vibration can be attributed to the 1381 cm^−1^. Further, by increasing the concentration of carbon in the 0.8 g sample, another peak at 1131 cm^−1^ was witnessed, that correlates to the C–O stretching vibrations or C–O–C epoxy vibrations. It can also be noticed that all the samples exhibited a broad peak at 3400 cm^−1^ due to the vibration of OH group and adsorption of water^25^.

### Brunauer–Emmett–Teller surface area studies

To investigate the surface area and pore volume of the 3D-F-KT nanostructures, Brunauer–Emmett–Teller technique was used, and the associated N_2_ adsorption–desorption isotherm studies were carried out at 77 K (Fig. 2b). A clear type-IV isotherm curve indicates the mesoporous nature of the material with an average pore diameter of 3.10 nm. Similarly, the specific surface area and pore volume were found to be 3.74 m^2^ g^−1^ and 0.009 cm^3^ g^−1^ respectively. The surface area has also contributed significantly to the enhanced photocatalytic activity of the 3D-F-KT nanostructures.

The mesoporous and type-IV isotherm nature of 0.2 and 0.8 g concentration of areca seed powder 3-D-F-KT was confirmed by BET analysis which is depicted in Fig-S1 Supporting information. The surface area contributes significantly to increase the photocatalytic activity of 3-D-F-KT nanostructures. As one can see the BET graphs of all areca seed concentrations i,e 0.2, 0.4, 0.8 g, the decrease in the surface area of 0.2 g (1.5 m^2^ g^−1^) and 0.8 g (0.5 m^2^ g^−1^) concentrations leads to the poor performance than 0.4 g concentration of areca seed powder 3-D-F-KT nanostructures in photocatalytic activity. In addition to this, the pore volume and pore diameter of 0.2 g and 0.8 g concentration of areca seed powder 3-D-F-KT was found to be 0.0029, 0.0006 cm^3^ g^−1^ and 5.4 nm, 7.8 nm respectively.

### X-ray photoelectron spectroscopy studies

XPS analysis was used to determine the elemental valence state of the material. As illustrated in Fig. 2c, survey of KTaO_3_ shows the presence of K, Ta and O and there different states. The peak of C 1*s* at 285.1 eV is caused by adventitious hydrocarbon in the XPS instrument^19^. Further, Fig. 2d revealed two peaks which are attributed to Ta 4f_7/2_ and Ta 4f_5/2_ at 26.15 and 27.34 eV respectively and it was evident that Ta existed as Ta^5+^ in KTaO_3_. Two peaks for K 2*p* were seen at 291.1 and 293.9 eV (Fig. 2e), corresponding to K 2*p*_3/2_ and K 2*p*_½_ respectively. The peak (Fig. 2f) at 531.27 eV for O 1*s* was due to O^2−^ which came from KTaO_3_ and another at 533.9 eV corresponds to the hydroxyl group. In addition, all different states of 0.2 g and 0.4 g concentration of areca seed powder 3D-F-KT NPs were given in Fig-S3 Supporting Information.

### Scanning electron microscopy (SEM)

**Figure 3:**
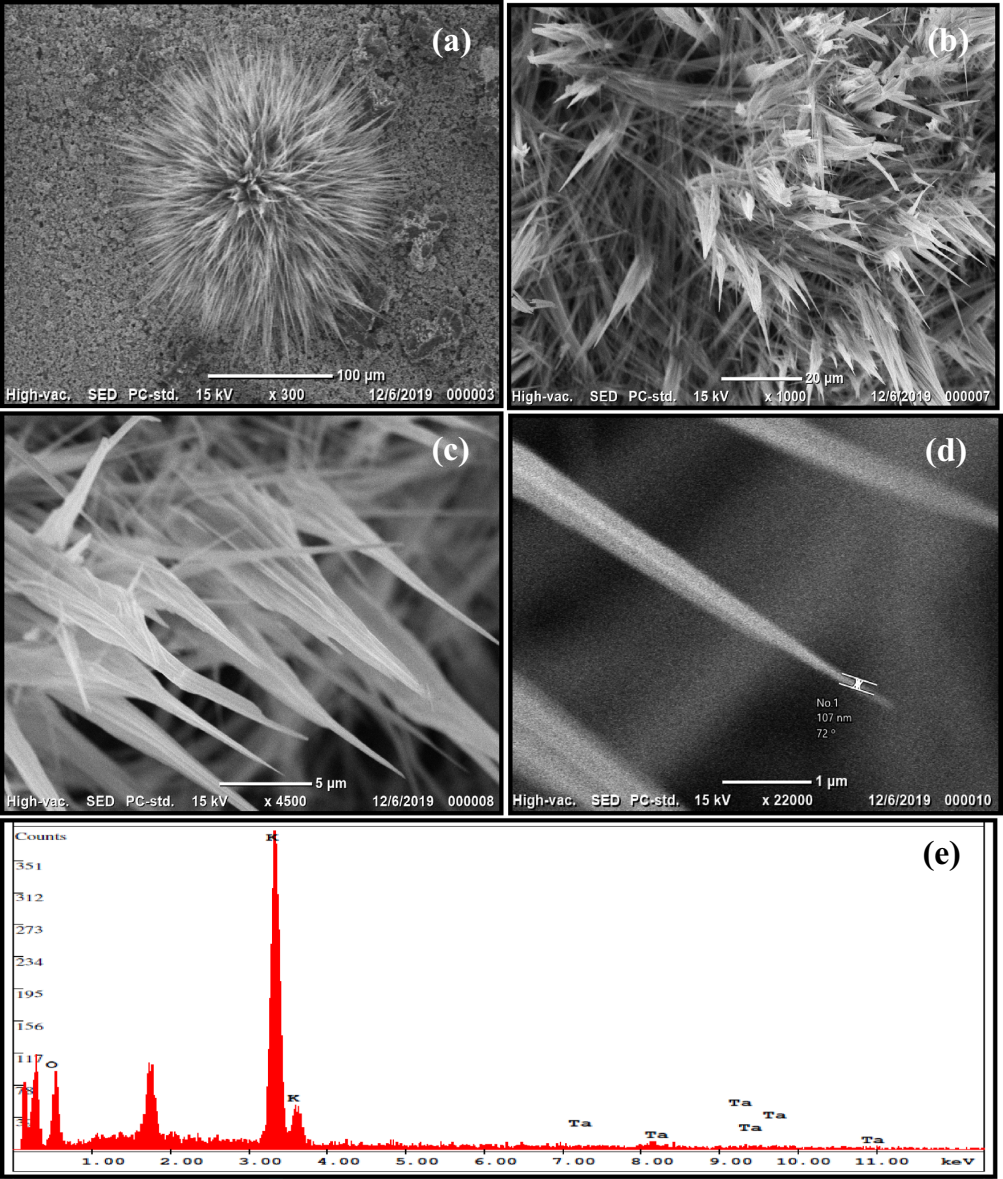
(**a**) 3D flowerlike structure observed at 100 µm range. (**b**, **c**) Bunch of flower petals showing sharp edges. (**c**) A needle embedded within single petal recorded at 1 µm range of (0.4 g concentration of areca seed powder 3D-F-KT).

The morphology of the 3D-F-KT nanostructures was studied using a scanning electron microscope (Neo-Scope JCM-6000PLUS system) and their representative images are shown in Fig. 3. As observed from the SEM images, the synthesized material KTaO_3_ has a well-developed flower like 3D structure with different sizes. It is the first time that flower-shaped 3D-KTaO_3_ nanostructures were synthesised by combustion method and are being reported. The distribution of elements and percentage composition of K, Ta, O in 0.4 g concentration of areca seed powder 3-D-F-KT was analysed and confirmed by elemental mapping (Fig-S2 Supporting information).

### Transmission electron microscope (TEM)

**Figure 4:**
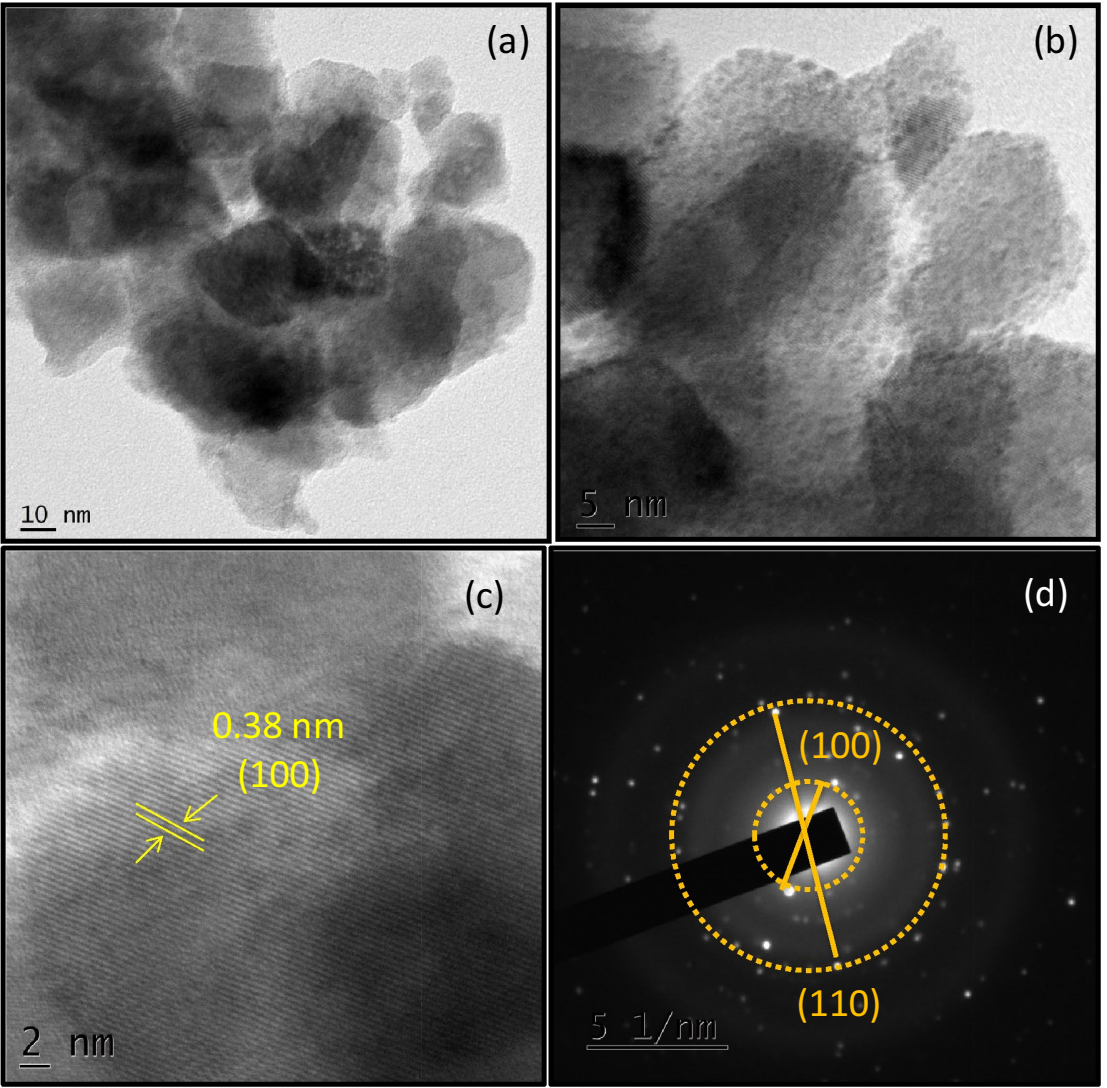
(**a**,**b**) TEM images with different magnifications (**c**) d-spacing value well matched with 100 XRD plane (**d**) SAED pattern showing 2 bright fringes of 3D-F-KT NPs (0.4 g concentration of areca seed powder 3D-F-KT).

The TEM (JEOL-JEM 2100 system) images of the nanostructures are shown in Fig. 4a,b. The d-spacing value was found to be 0.38 nm as shown in Fig. 4c. This matches well with the (100) plane obtained from the XRD analysis. Figure 4d shows the SAED pattern with 2 bright fringes and corresponds to the XRD planes (100) and (110).

### Photocatalytic degradation

**Scheme 1:**
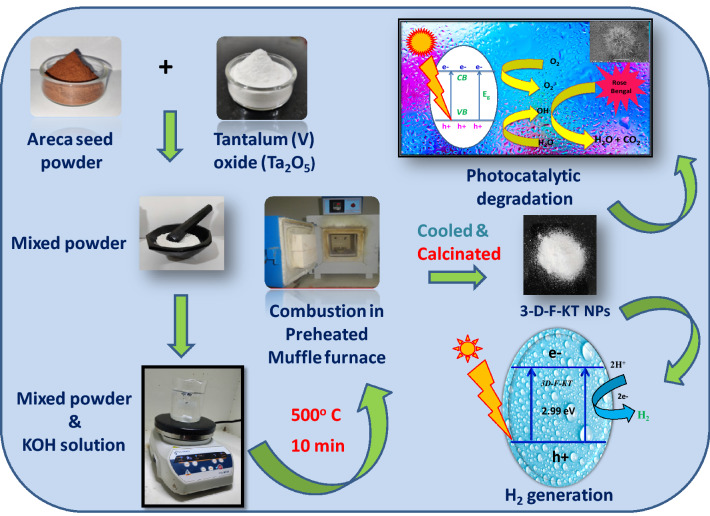
Overall synthesis process and schematic representation of visible light degradation of rose Bengal dye and H_2_ generation.

**Figure 5:**
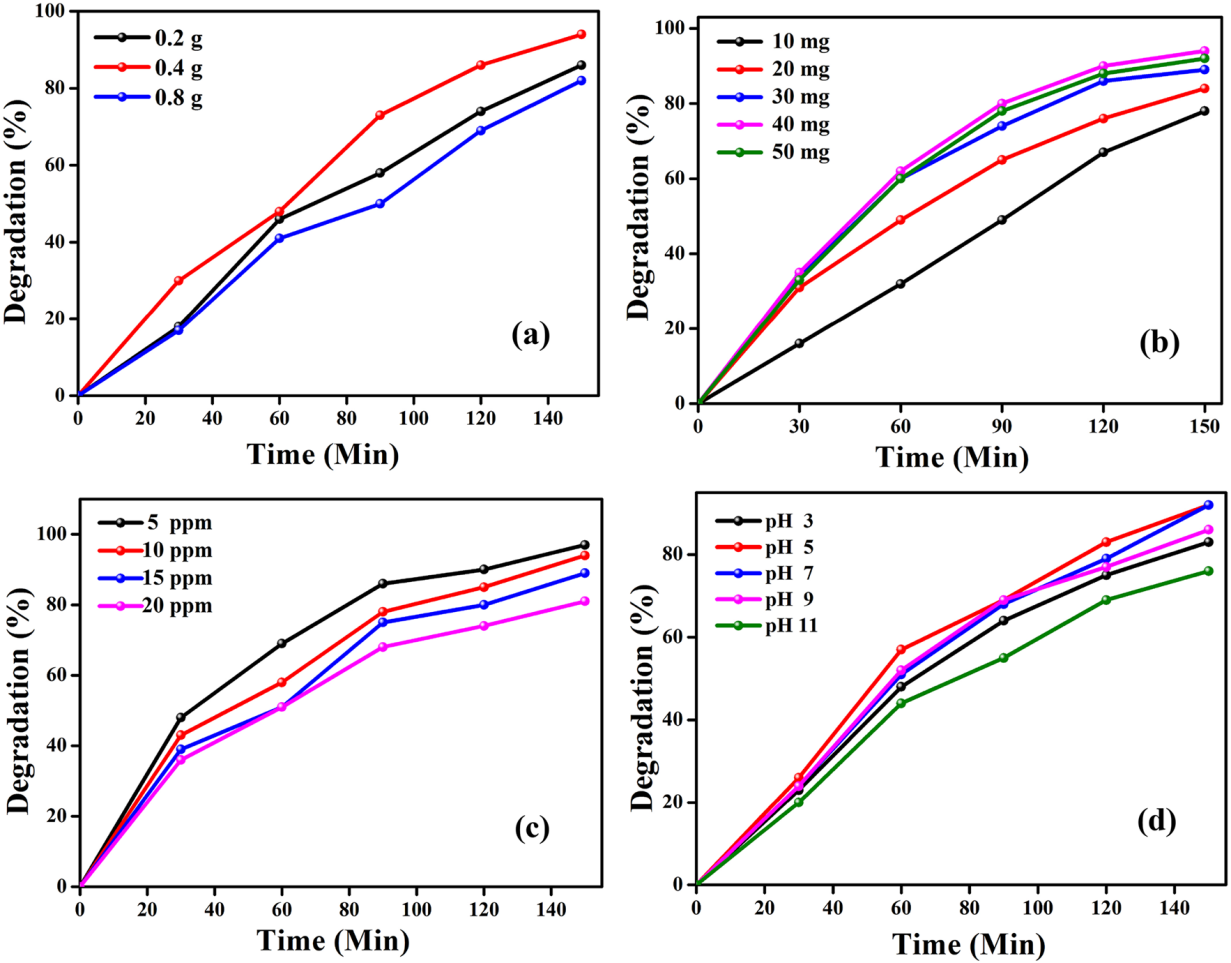
(**a**) Degradation plot of rose Bengal dye with 3 different fuel ratios of 3D-F-KT NPs (**b**) Photocatalytic activity at different dye concentrations using 0.4 g catalyst (**c**) Photocatalytic activity of 5 ppm RB dye with different catalytic load of KT NPs (**d**) Effect of pH on photocatalytic activity of 3D-F-KT NPs.

The photocatalytic activity of 3D-F-KT nanostructures with different concentration (0.2, 0.4, 0.8 g) was examined using rose bengal dye in the presence of visible light irradiation (λ > 420 nm) and a schematic representation depicting the process has been shown in Scheme 1. Initially, to observe the degradation efficiency in the absence of visible light, photolysis was conducted in a dark condition. After 30 min of the reaction time, no degradation was observed. Later, the experiment was carried out by collecting the reaction mixture for every 30 min to check the rate of dye degradation. As it can be observed from Fig. 5a, 0.4 g of the fuel concentration showed appreciable degradation efficiency (94.12%) compared to other fuel concentrations. A comparative analyse of the degradation property of KTaO_3_ nanostructures with different dyes and pollutants were made and their data is given in Table 1. In this table details of various KTaO_3_ composites and bare KTaO_3_ materials were given, they have been tested with the degradation of organic and dye pollutants like phenol, toluene, rhodamine B and Methylene blue with acceptable degradation efficiency. Moreover, degradation performance 3D-F-KT against rose Bengal dye proves the outstanding ability of material in contrast to previously reported photocatalysts.

**Table 1:** Literature survey with degradation of different pollutants by KTaO_3_ and its composites.

Material	Source of light	Dye	% of degradation	Time (min)	References
30 wt% rGO-KTaO_3_	Visible	Phenol	43	60	^25^
0.5Au/1.5Pt-KTaO_3_	Visible	Phenol	14.75	90	^32^
2.0 Rh-KTaO_3_	Visible	Toluene	41.98	60	^32^
KTaO_3_ + CdS + MoS_2_ (10:5:1)	LED	Toluene	60	60	^33^
KTaO_3_	LED	Toluene	64	60	^33^
KTaO_3_-CdS(10:1)	UV–Vis	Phenol	59	60	^33^
54 wt%BiOI/KTaO_3_	Visible	Rhodamine B	98.6	30	^19^
N-KTaO_3_	Visible	Methylene blue	98.3	240	^34^
KTaO_3_	Visible	Rose bengal	94.12	150	Present work

#### Effect of catalytic load

It is well-known that the amount of photocatalyst significantly affects the degradation rate of dyes. Hence, to find out the optimum catalytic load, different amounts of the catalyst (10, 20, 30, 40 mg) were used with rose bengal dye at pH 7. The effect of different amount of the photocatalyst (KTaO_3_ nanostructures) on the degradation rate has been shown in Fig. 5b. Upon increasing the catalytic load, the degradation rate also increased up to a certain amount and thereafterno effect was observed. This may be due to the fact that as the amount of photocatalyst increased, the number of active sites available on the surface of KTaO_3_ nanostructures also increases with an increase in the exposed surface area. However, after a certain weight i.e., 40 mg, there was no increase in the specific surface area and hence, the active sites. This was considered to be the saturation point, above which not much of photocatalytic degradation was observed, as adding the photocatalyst beyond this point would allow it to settle at the bottom of the tube. Further, the formation of turbidity in the reaction mass indicates the fall of degradation rate. Therefore, the optimum weight of the photocatalyst for the effective degradation of rose bengal dye was found to be 40 mg.

#### Effect of dye concentration

Figure 5c shows the effect of different concentrations of rose bengal dye on the photocatalytic performance of 3D-F-KT nanostructures. With a fixed catalytic load of 40 mg at neutral pH, the concentration of rose bengal dye was varied in terms of 5, 10, 15, 20 ppm per 100 ml of the reaction mixture. It was concluded that the photodegradation efficiency of rose bengal dye was inversely proportional to its concentration, which means that when the dye concentration was more, its photocatalytic degradation rate decreased at a fixed amount of catalyst. This may be attributed to the fact that the dye itself acts as an obstacle for the intensity of incident light and thus, affects the path length of the incoming light. Hence, it can be concluded that at 5 ppm dye concentration, a degradation efficiency of 90% was observed.

#### Effect of pH

As shown in Fig. 5d, pH plays an important role in varying the rate of photocatalytic degradation of dyes. The effect of pH on the degradation of rose bengal was studied by keeping the amount of catalyst and the dye concentration constant. In a solution having pH 3, the surface of the photocatalyst and the dye molecules become positively charged so that the dye molecule and the catalyst will repel each other. Thus, the catalytic reaction on the surface of the material takes place towards the smaller extent. Whereas a gradual decrease in the degradation rate was observed in alkaline solutions. This is due to the electrostatic repulsion between the negatively charged surface of the catalyst and the anionic rose bengal dye. So, the optimum pH value for the excellent degradation of rose bengal was found to be pH 5^26^.

#### Mechanism of dye degradation

The generation of electrons in the conduction band and holes in the valence band takes place when the semiconductor nanostructures absorb light energy from an external source. The possible mechanism is show below in Eq. (4).

4 $${\text{KTaO}}_{3} + {\text{h}}\nu \to {\text{KTaO}}_{3} ({\text{e}}^{ - } ({\text{CB}}) + {\text{h}}^{ + } ({\text{VB}}))$$

Water reacts with the generated holes at the valence band to give OH^•^ which is a powerful oxidising agent to attack the nearest dye molecule as shown in Eq. (5).

5 $${\text{H}}_{2} {\text{O}} + {\text{h}}^{ + } ({\text{VB}}) \to {\text{OH}}^{ \cdot } + {\text{H}}^{ + }$$

Later, oxygen reacts with the generated electrons at the conduction band to give an anionic superoxide radical (O_2_^•^‾)

6 $${\text{O}}_{2} + {\text{e}}^{ - } ({\text{CB}}) \to {\text{O}}_{2}^{ \cdot - }$$

The recombination of the electron and the hole was thus reduced by the formation of a superoxide ion on 3D-F-KT surface and thus, maintains the neutrality of electrons. The generated O_2_^•^‾ is protonated to produce H_2_O_2_ and therefore, the OH^•^ radical remains at last. Finally, the conversion of the hazardous dye (RB) into CO_2_ and water takes place efficiently (7–10).

7 $${\text{O}}_{2}^{ \cdot - } + {\text{H}}^{ + } \to {\text{HOO}}^{ \cdot }$$

8 $$2{\text{HOO}}^{ \cdot } \to {\text{H}}_{2} {\text{O}}_{2} + {\text{O}}_{2}$$

9 $${\text{H}}_{2} {\text{O}}_{2} \to 2{\text{OH}}^{ \cdot }$$

10 $${\text{Dye}} + {\text{OH}}^{ \cdot } \to {\text{CO}}_{2} + {\text{H}}_{2} {\text{O}}$$

#### Electrochemical tests and kinetics of photocatalytic degradation

**Figure 6:**
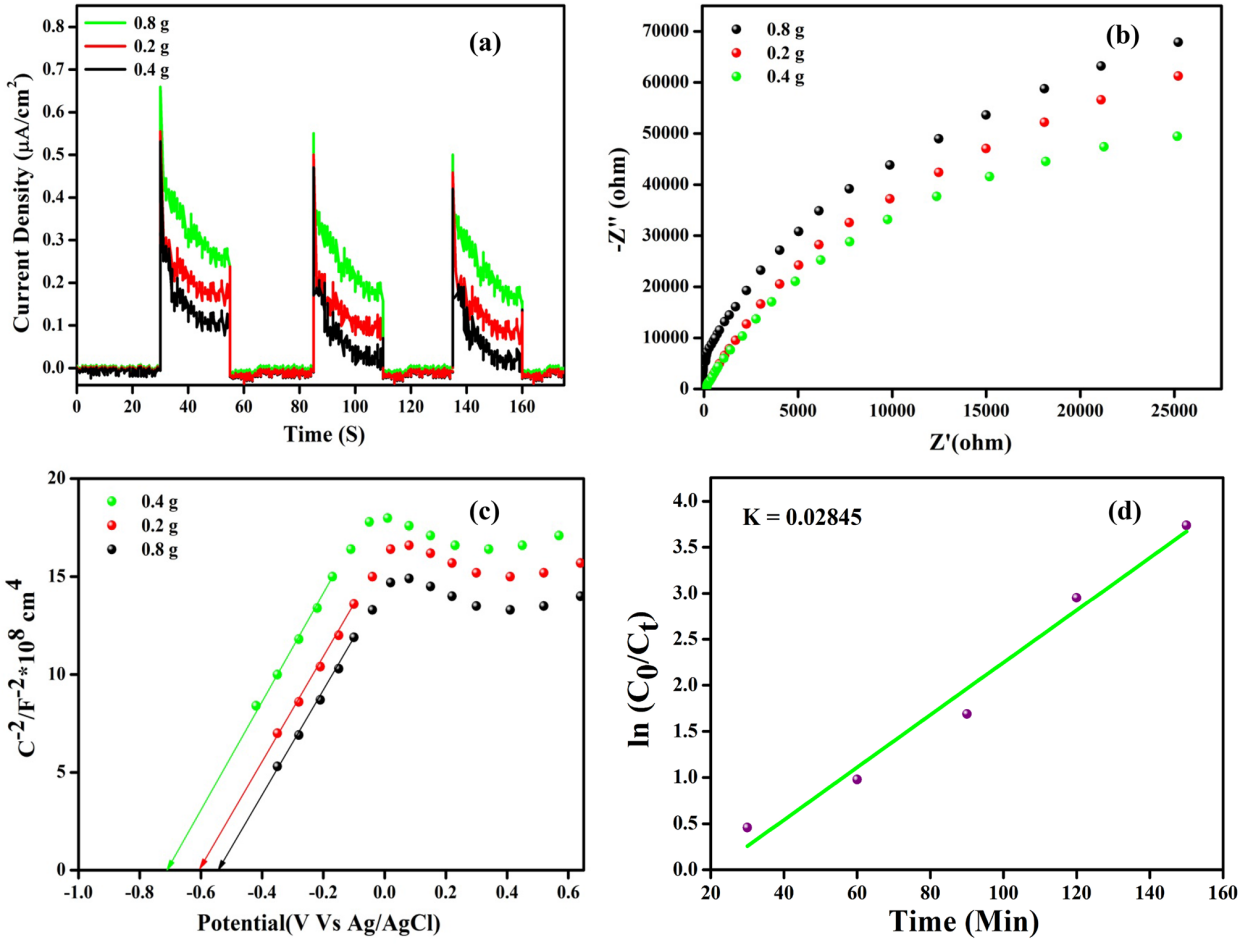
(**a**) 0.4 g concentration of areca seed powder 3-D-F-KT showing more separation of photo induced carriers by exhibiting higher photocurrent value than other two concentrations. (**b**) Very less arc radius recorded for 0.4 g concentration of areca seed powder 3-D-F-KT by EIS method. (**c**) Mott–Schottky plots showing nature of semiconductors. (**d**) Rate constant plot for 0.4 g concentration of areca seed powder 3D-F-KT.

Photoelectrochemical studies were performed to better study the separation and transfer of photo-induced electrons and holes. The photocurrent–time curves of 3-D-F-KT samples under visible light irradiation are shown in Fig. 6a. The photocurrent intensity was ordered as follows: 0.4 g > 0.2 g > 0.8 g concentrations of areca seed powder 3-D-F-KT, which was consistent with the degradation profiles. In general, a higher photocurrent indicates the more separation of photoinduced carriers.

0.4 g concentration of areca seed powder 3-D-F-KT photocatalyst has the least arc radius as shown in Fig. 6b, suggesting the shortest interfacial charge-transfer resistance, which results in good electron and hole separation efficiency^19^. The improved separation efficiency owing to areca seed powder doping can be attributed to the high internal electric field (IEF), which allows for the separation of electrons and holes immediately after their formation.

The Mott–Schottky test was used to confirm the semiconductor nature of 3 different concentrations (0.2, 0.4, 0.8 g) of areca seed powder 3-D-F-KT nanostructures. As illustrated in Fig. 6 c, 3-D-F-KT has been demonstrated to be an n-type semiconductor with a positive slope on the Mott–Schottky curve. The flat band potentials (V_fb_) of all 3 samples were determined by intersecting the tangent drawn to Mott-schottky curves with potential axis. The V_fb_ value is found to be more negative for 0.4 g concentration of areca seed powder 3-D-F-KT than 0.2 and 0.8 g, which indicates the existence of a very small barrier for charge transfer. In general, more negative V_fb_ value means stronger is the photoelectrochemical performance of semiconductors.

To investigate the positions of valence band (E_VB_) and conduction band (E_CB_) of all 3 different concentrations of areca seed powder 3-D-F-KT samples, we adopted the formula: E_VB_ = X–E_0_ + 0.5 E_g_ and E_CB_ = E_VB_–E_g_, where X implies the absolute electronegativity of material (depending upon individual composition of atoms), E_0_ denotes the free electrons energy on hydrogen scale (i.e. 4.5 eV vs. NHE) and E_g_ is the band gap energy of semiconductor. The determined E_CB_ and E_VB_ values for 0.2, 0.4 and 0.8 g concentration of areca seed powder 3-D-F-KT were found to be (− 0.57 and 2.41 eV), (− 0.575 and 2.415 eV) and (− 0.565 and 2.405 eV) respectively, where X value is calculated as 5.42 eV^19^.

The kinetic study was carried out using Langmuir–Hinshelwood model for the rose bengal dye over 3D-F-KT nanostructures. The straight line obtained from the kinetic model indicates the dye removal over KTaO_3_ and follows a pseudo-first-order kinetics as shown in Fig. 6d. The observed slope value (k) from the plot was found to be 2.8 × 10^–2^ min^−1^ by using the formula ln (C_o_/C_t_) = kt, where C_o_ represents the initial concentration of the dye at t = 0 and C_t_ is related to the final concentration after every 30 min.

#### Effect of scavengers and co-existing ions

**Figure 7:**
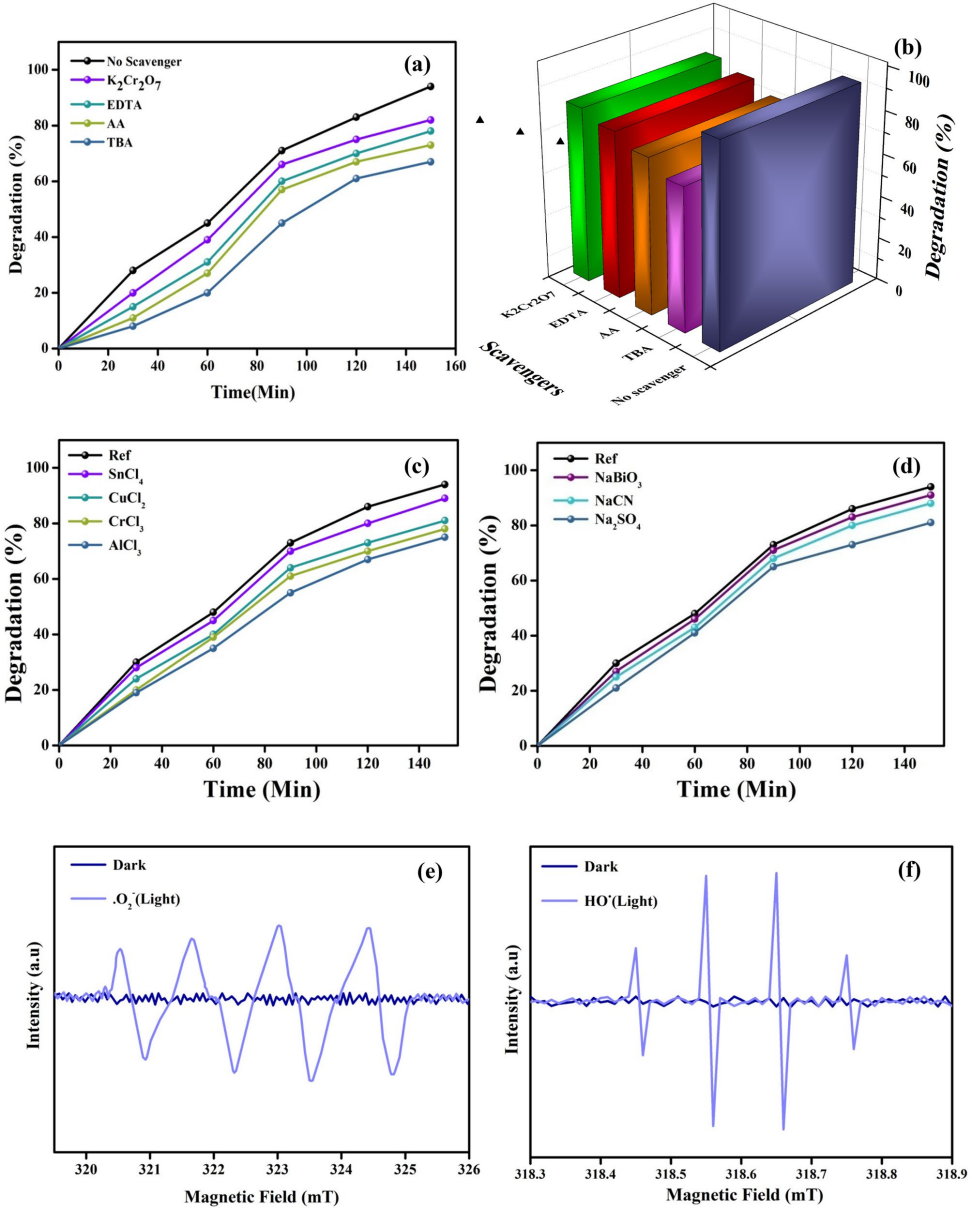
(**a**) Degradation curve with time of influence of scavengers on photodegradation of rose Bengal and (**b**) Bar graph showing major role of OH radical in degradation process under visible light irradiation using 0.4 g concentaration of areca seed powder 3-D-F-KT. Effect of co-existing ions like cations (**c**) and anions (**d**) on photocatalytic degradation of Rb. (**e**,**f**) Detection of active radical species using ESR spectral analysis, showing HO• and O_2_^•−^ are the two reactive species leads degradation of Rb dye.

The process of photocatalysis under visible light irradiation was examined using scavenging experiments by trapping the active species produced at the time of reaction. Here, tertiary-butyl alcohol (TBA) and potassium dichromate (K_2_Cr_2_O_7_) were used as the scavengers for OH^•^ and e^−^ respectively, while ascorbic acid (AA) and ethylenediaminetetraacetic acid (EDTA) were adopted as the scavengers for O_2_^•−^ and h^+^ respectively. Figure 7a,b represents the degradation efficiency of rose bengal dye using 40 mg of 3D-F-KT without adding any scavengers. Apparently, the percentage degradation of the rose bengal dye did not exhibit much change after the addition of K_2_Cr_2_O_7_ and EDTA, which implies that e^−^ and h^+^ played a negligible role in the degradation process. However, with the addition of TBA or AA, the degradation rate of the rose bengal dye reduced, and only 67.86% or 73.13% degraded after 120 min of the reaction. The obtained result shows that OH^•^ was the primary reactive species in the photocatalytic degradation of the rose bengal dye.

The effects of various cations (Cu^2+^, Al^3+^, Cr^3+^ and Sn4^+^) and anions (BiO^3−^, SO_4_^2−^, CN^−^) on this photocatalytic process are investigated. As illustrated in Fig. 7c,d. The degradation rate appeared to reduce when 100 mg L^−1^ of cations and anions were added, however the effect of anions was minimal. The explanations are most likely related to redox reactions of ions on the catalyst's surface. BiO^3−^ and CN^−^ anions compete with dye as they oxidise at the catalyst surface. These anions, however, are protonated and neutralised because the reaction occurred at pH 5. As a result, they are unable to compete with dye for photocatalytic oxidation.

#### Radical detection

The electron spin resonance (ESR) technique was utilised to detect the radicals formed during the photocatalytic process and their function in photocatalysis. 5,5-Dimethyl1-Pyrroline-N-Oxide (DMPO) was used as the radical capture agent. The ESR results demonstrated that superoxide (O_2_^−^•), and hydroxyl (HO•) radicals were formed during the Vis/3-D-F-KT based photocatalysis system, and these two radicals being primarily responsible for dye degradation. Figure 7e,f shows clearly that the dominating radical in the photocatalysis process is HO•, whereas O_2_^−^• plays a supporting role. ESR spectra also demonstrated the effect of light on the activation of radicals generated by photocatalyst. Initially, no radicals were generated in the dark, but as the catalytic system was exposed to light, the production of radicals increased exponentially with time.

#### Recycling experiment

**Figure 8:**
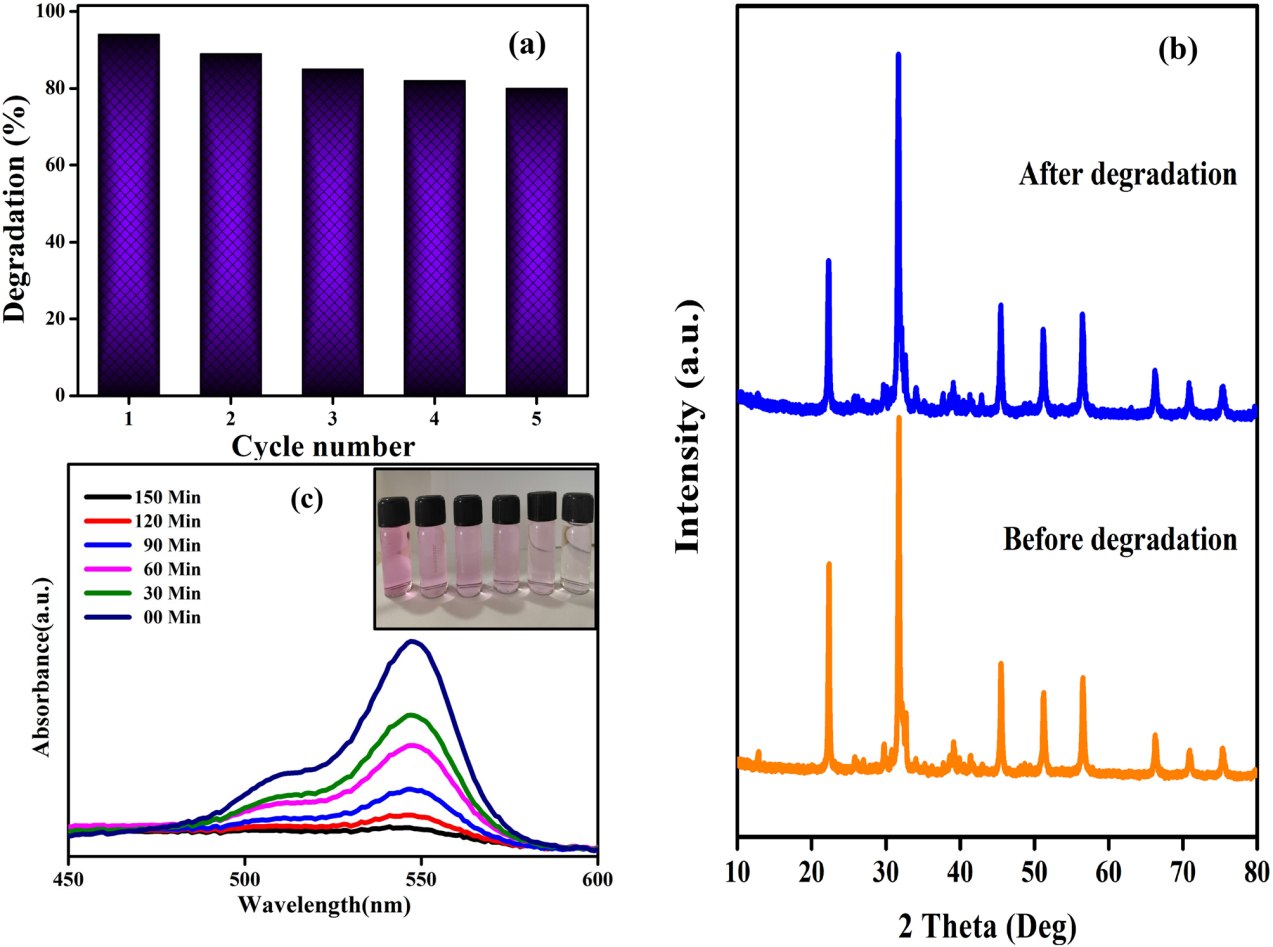
(**a**) Reusability over 5 cycles is achieved with excellent degradation efficiency and (**b**) XRD spectrum showing no change in peaks intensity-before and after degradation of 3D-F-KT (0.4 g concentration of areca seed powder). (**c**) UV–Vis spectra of 0.4 g concentration of areca seed powder 3D-F-KT along with time (Inset: Picture showing effective degradation of Rb dye).

The reusability and photostability of 3D-F-KT nanostructures were analysed using recycling experiments under the irradiation of visible light for 5 cycles as shown in Fig. 8a. The photoreaction mixture was collected after every cycle, followed by centrifugation and filtration. The collected residue was washed with distilled water and was later recovered. The recovered material residue was reused for the subsequent degradation experiment as earlier. The degradation efficiency of the rose bengal dye decreased from 94.12 to 80.26% for the 1st and 5th cycles respectively. However, a loss in efficiency of only 13.86% was observed even after the 5th cycle, which shows that KTaO_3_ nanostructures had an excellent photostability. Meanwhile, Fig. 8b represents the XRD spectrum of 3D-F-KT nanostructures before and after the degradation process. However, no differences in the peaks were found and the structure of the material appeared to be stable even after the degradation of the rose bengal dye. In adition to this, UV–Vis spectra of 0.4 g concentration of areca seed powder 3D-F-KT along with time are depicted in Fig. 8c.

#### Detection of hydroxyl radicals

**Figure 9:**
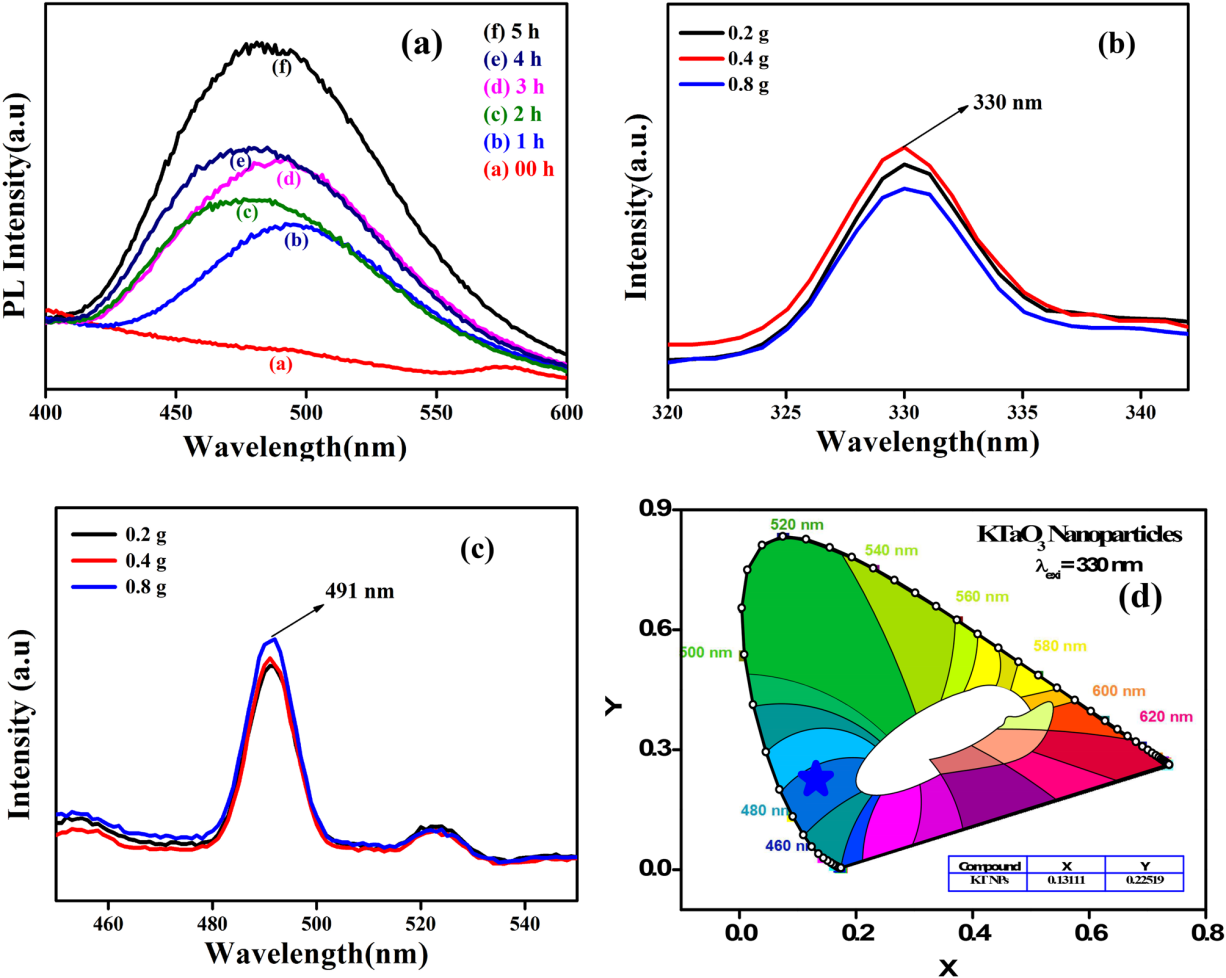
(**a**) PL spectra showing the presence of large amount of OH radicals by forming 7-hydroxyl coumarin from coumarin upon increasing the time. (**b**) PL excitation peak (**c**) emission peak (**d**) CIE diagram showing the emission of blue colour of 3D-F-KT NPs prepared by different concentrations of areca seed powder.

The rate of formation of OH radicals can be found by a simple and effective photoluminescence (PL) technique by taking Coumarin as an investigator. During the degradation process, OH radicals are the most important reactants; it reacts with the coumarin molecule to produce 7-hydroxyl coumarin. In this study, 50 mg of 3D-F-KT nanostructures was dispersed in 100 ml of aqueous 0.5 mM coumarin and were air bubbled for 15 min to understand the adsorption–desorption equilibrium in the dark condition. Furthermore, 300 W tungsten light source was used as an illuminator. 5 mL of the reaction mixture was drawn at every 30 min to measure the photoluminescence spectrum using the Agilent Technologies Cary Eclipse-60 Spectrophotometer. From Fig. 9a, it is evident that the intensity of the peaks in the PL spectra was directly proportional to the time of reaction. The variation in peak positions at different delay durations implies the involvement of more than one excited state. Peak shifts are ascribed to changes in the emission intensity ratio of the surface trapping and excitonic states on the conduction band edge over time^27^. This provides stronger evidence to show the formation of OH radicals on the surface of the photocatalyst. Later, the OH radical production increases with time.

### Photoluminescence studies

The recombination efficiency of the photogenerated free charge carriers can be predicted by photoluminescence studies (PL). At room temperature, the excitation and emission spectra of 3D-F-KT nanostructures were recorded and are shown in Fig. 9b,c respectively. The emission peak of 491 nm was observed at an excitation wavelength of 330 nm^25,28^. The samples prepared by adding 0.2 and 0.4 g of the fuel showed lower PL intensities as compared to 0.8 g of the sample. This could lead to the fact that, more intensity of the emission peak indicates a higher recombination of the photogenerated electron and hole pairs, and therefore, lesser is the photocatalytic activity. It can also be seen that nanostructures synthesised using 0.2 and 0.4 g of the fuel gave almost similar photoluminescence peaks and were found have the most efficient separation of charge carriers. The colour of the emission spectra is shown in Fig. 9d and it is clear that the electrons jumped from the conduction band to the valence band by losing some amount of energy and hence, a higher emission wavelength at 491 nm and a lower excitation at 330 nm were observed. Meanwhile, it can be concluded that the emission of blue colour at 491 nm is as per the Commission International de I’Eclairage (CIE) 1931 chromaticity diagram^28^.

## Photocatalytic H_2_ generation

**Figure 10:**
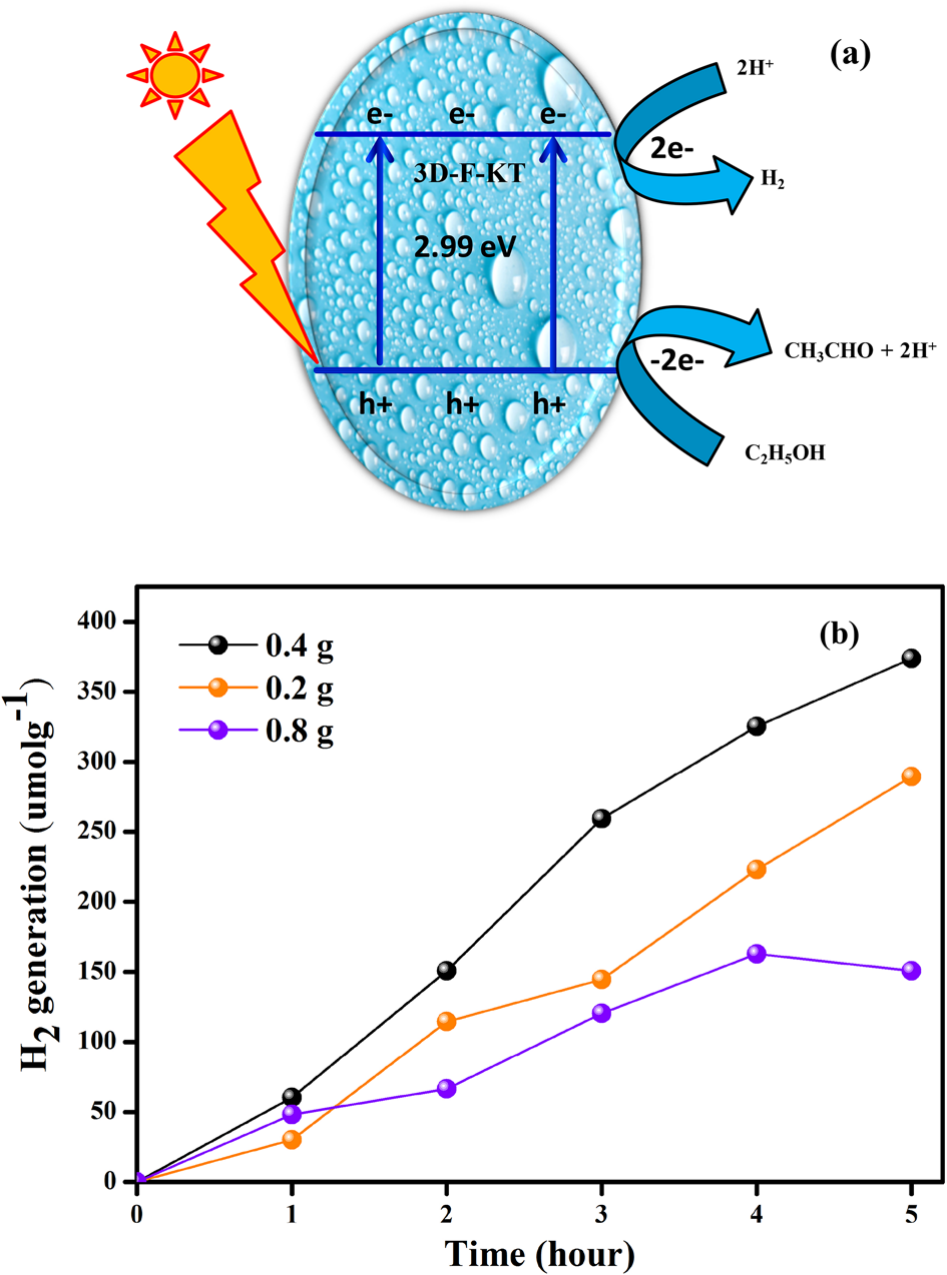
(**a**) Schematic representation of photocatalytic H_2_ production under UV–visible light irradiation. (**b**) 3D-F KT prepared by 0.4 g concentration of areca seed powder shows higher H_2_ generation than other 2 concentrations.

The photocatalytic H_2_ generation of the synthesised 3D-F-KT nanostructures was analysed using a 400 W Xenon lamp (UV–Vis light source). If the energy of the conduction band was negative compared to the reduction potential value H^+^/H_2_ (0 V vs. NHE), and the energy of the valence band has a higher positive value than the oxidation potential O_2_/H_2_O (1.23 V vs. NHE), then the photocatalyst should satisfy the condition for water splitting. On irradiating the energy higher than the band gap energy of KTaO_3_ photocatalyst, the excitation of electrons from valence to conduction band occurs by leaving holes in the lower energy level. To avoid the electron–hole recombination, the sacrificial agent consumes the produced holes from the valence band and thereby, results in an increased H_2_ production. The factors for the photocatalyst to show good hydrogen generation are crystallinity, band gap, thickness of pore wall, particle size, hydrophilic group on the catalyst surface and the surface area. As depicted in Fig. 10b, the hydrogen generation of 3D-F-KT (0.4 g concentration of areca seed powder) photocatalyst was found to be 374 µmol g^−1^ for 5 h. The photocatalytic activity of this material was based on factors like crystallinity, surface area, particle size, thickness of pore wall, number of hydrophilic groups on the catalyst surface and also the existence of active sites^29^. The schematic representation of the photocatalytic H_2_ generation is given in Fig. 10a. Moreover, 3D-F-KT nanostructures with 0.4 g fuel show a higher H_2_ generation value than the other two samples (0.2 and 0.8 g). Here, the higher H_2_ generation was due to the more hydroxyl groups on the surface of the material^30,31^.

## Conclusion

The multifunctional property of potassium tantalate is the key factor behind its large application in various fields. In this way, we have successfully prepared novel 3D-F-KT nanostructures using areca seed powder as a green fuel by combustion method. All 3D-F-KT samples with different fuel ratios show an excellent photocatalytic activity under the irradiation of visible light. The prepared material shows an excellent degradation efficiency of up to 94.12% in a short time under visible light irradiation and also appreciable amount of H_2_ is generated with a good potential of material. In addition, the photoluminescence (PL) and all other electrochemical studies proved that there was very less recombination of charge carriers during the reaction. The recycling experiment also shows the excellent durability of the photocatalyst. Finally, by observing the obtained results, it can be inferred that KTaO_3_ can be adopted as a promising and an efficient material for both wastewater treatment and H_2_ generation.

## Supplementary Information

Supplementary Information.
