# Supplementary material for: Novel 3D-flower shaped KTaO3 perovskite for highly efficient photocatalytic and H2 generation ability
Source: Sci Rep. 2022 Jun 24;12:10776. doi: 10.1038/s41598-022-14590-3 (PMC9232656; doi:10.1038/s41598-022-14590-3)
Supplement: Supplementary file 1 — Supplementary Information. [file 41598_2022_14590_MOESM1_ESM.doc]

**Novel 3D-Flower Shaped KTaO_3_ Perovskite for Highly Efficient Photocatalytic and H_2_ Generation Ability**

H. N. Sumedha ^1,2^, M. Shashank^3,4^, Sergio R. Teixeira^5^, B.M. Praveen^1*^, G. Nagaraju ^3*^

*^1^Department of Chemistry, Institute of Engineering and Technology, Srinivas University, Mukka, Mangalore, Karnataka 574146, India.*

*^2^Centre for Incubation, Innovation, Research and Consultancy (CIIRC), Jyothy Institute of Technology, Thataguni, Bengaluru - 560082, Karnataka, India.*

*^3^Energy Materials Research Laboratory, Siddaganga Institute of Technology (Affiliated to VTU, Belagavi), Tumakuru, Karnataka 572103, India.*

*^4^Department of Studies and Research in Industrial Chemistry, School of Chemical Science, Kuvempu University, Shankaraghatta, Karnataka 577451.*

*^5^Laboratory of Thin Films and Nanostructure Fabrication (L3Fnano), Institute of Physics, Universidade Federal do Rio Grande do Sul, UFRGS, Brazil.*

**BET surface area:**

**Fig-S1**: BET plots of 0.2g concentration of areca seed powder 3-D-F-KT (left) and 0.8g concentration of areca seed powder 3-D-F-KT (right). Both showing comparatively less surface area than 0.4 g concentration of areca seed powder 3-D-F-KT (BET plot depicted in main manuscript (Fig-6))

**Elemental mapping:**

**Fig-S2:**(a-d) Elemental mapping shows distribution of elements in 0.4 g concentration of areca seed powder 3-D-F-KT nanostructures.(e) Percentage compositions of K, Ta and O in 0.4 g concentration of areca seed powder 3-D-F-KT.

**Fig-S3**: XPS spectra showing different valence states of 3D-F-KT nanoparticles. (a) Ta 4f_5/2_ and Ta 4f _7/2_. (c) K 2p_1/2_ and _3/2_ (e) O 1s of 0.2 g concentration of areca seed powder 3D-F-KT. (b) Ta 4f_5/2_ and Ta 4f _7/2_. (d) K 2p_1/2_ and _3/2_ (f) O 1s of 0.8 g concentration of areca seed powder 3D-F-KT.
